# Agonist-activated glucagon receptors are deubiquitinated at early endosomes by two distinct deubiquitinases to facilitate Rab4a-dependent recycling

**DOI:** 10.1074/jbc.RA120.014532

**Published:** 2021-01-13

**Authors:** Suneet Kaur, Yuqing Chen, Sudha K. Shenoy

**Affiliations:** 1 Division of Cardiology, Department of Medicine, Duke University Medical Center, Durham, North Carolina, USA; 2 Department of Cell Biology, Duke University Medical Center, Durham, North Carolina, USA

**Keywords:** G protein-coupled receptor, ubiquitination, endocytosis, glucagon, ubiquitin-specific protease, deubiquitylation (deubiquitination), G protein-coupled receptor (GPCR), receptor recycling

## Abstract

The glucagon receptor (GCGR) activated by the peptide hormone glucagon is a seven-transmembrane G protein–coupled receptor (GPCR) that regulates blood glucose levels. Ubiquitination influences trafficking and signaling of many GPCRs, but its characterization for the GCGR is lacking. Using endocytic colocalization and ubiquitination assays, we have identified a correlation between the ubiquitination profile and recycling of the GCGR. Our experiments revealed that GCGRs are constitutively ubiquitinated at the cell surface. Glucagon stimulation not only promoted GCGR endocytic trafficking through Rab5a early endosomes and Rab4a recycling endosomes, but also induced rapid deubiquitination of GCGRs. Inhibiting GCGR internalization or disrupting endocytic trafficking prevented agonist-induced deubiquitination of the GCGR. Furthermore, a Rab4a dominant negative (DN) that blocks trafficking at recycling endosomes enabled GCGR deubiquitination, whereas a Rab5a DN that blocks trafficking at early endosomes eliminated agonist-induced GCGR deubiquitination. By down-regulating candidate deubiquitinases that are either linked with GPCR trafficking or localized on endosomes, we identified signal-transducing adaptor molecule–binding protein (STAMBP) and ubiquitin-specific protease 33 (USP33) as cognate deubiquitinases for the GCGR. Our data suggest that USP33 constitutively deubiquitinates the GCGR, whereas both STAMBP and USP33 deubiquitinate agonist-activated GCGRs at early endosomes. A mutant GCGR with all five intracellular lysines altered to arginines remains deubiquitinated and shows augmented trafficking to Rab4a recycling endosomes compared with the WT, thus affirming the role of deubiquitination in GCGR recycling. We conclude that the GCGRs are rapidly deubiquitinated after agonist-activation to facilitate Rab4a-dependent recycling and that USP33 and STAMBP activities are critical for the endocytic recycling of the GCGR.

Glucagon is a peptide hormone secreted by the alpha cells of the islets of Langerhans in response to hypoglycemia. Glucagon exerts its effect by binding to the seven-transmembrane glucagon receptor (GCGR) and activating adenylyl cyclase via stimulatory heterotrimeric G protein (G_s_) coupling (1, 2). GCGR-induced phosphorylation cascades triggered by cAMP-dependent protein kinase A results in gluconeogenesis and glycogenolysis. The GCGR is an emerging target in anti-diabetic therapy, particularly in the development of GCGR/GLP-1R co-agonists, which are predicted to have additive effects on body weight reduction and glycemia (3, 4). Dysregulated GCGR trafficking and signaling are implicated in the development of pancreatic α-cell hyperplasia and pancreatic neuroendocrine tumors (5, 6, 7).

Glucagon also induces phosphorylation of the GCGR by the GPCR kinases (GRKs) 2, 3, and 5 and protein kinase Cα, promoting recruitment of the endocytic adaptor proteins β-arrestin 1 and β-arrestin 2, leading to clathrin-dependent internalization (8). GCGR interacts with a single transmembrane protein called receptor activity–modifying protein 2, which reduces the cell-surface expression and dampens cAMP activation by glucagon (9, 10, 11, 12). Despite these characterizations, the molecular mechanisms that regulate GCGR trafficking and plasma membrane recycling are not completely understood.

Ubiquitination of mammalian GPCRs, demonstrated initially for the β-adrenergic receptor and the CXCR4 chemokine receptor, has been identified as an important post-translational modification that directs the endocytic sorting and lysosomal degradation of internalized GPCRs (13, 14, 15). Expanding work in this area has revealed that ubiquitination of a GPCR can also provoke signal transduction: protease-activated receptor 1 (PAR1) traffics independently of PAR1 ubiquitination but requires PAR1 ubiquitination to trigger signaling cascades through p38 mitogen-activated protein kinases (16, 17). Although not extensive, published studies also point to a critical regulatory role for deubiquitinases that reverse protein ubiquitination in GPCR trafficking and signaling pathways (18, 19, 20).

Whereas the effect of receptor ubiquitination has been reported for more than forty different members of the superfamily of GPCRs (20), whether the GCGR is regulated by ubiquitination has remained unknown. Additionally, there are no detailed studies addressing ubiquitination of related class B subfamily of receptors, namely, glucagon-like peptide-1 (GLP-1) and GLP-2 receptors. On the other hand, glucose-dependent insulinotropic polypeptide (GIP) receptor has been reported to be ubiquitinated and down-regulated in isolated rat pancreatic islets upon prolonged exposure to high glucose concentration in culture medium (21). In this study, we have ascertained the effects of the endogenous agonist glucagon on GCGR ubiquitination and correlated them with the endocytic trafficking of the receptor. We have also generated a mutant GCGR that is defective in ubiquitination and compared its trafficking with the WT GCGR. Additionally, by systematically screening different deubiquitinases involved in trafficking pathways, we have linked the GCGR with its cognate deubiquitinating enzymes.

## Results

### Glucagon induces deubiquitination of the GCGR

**Figure 1:**
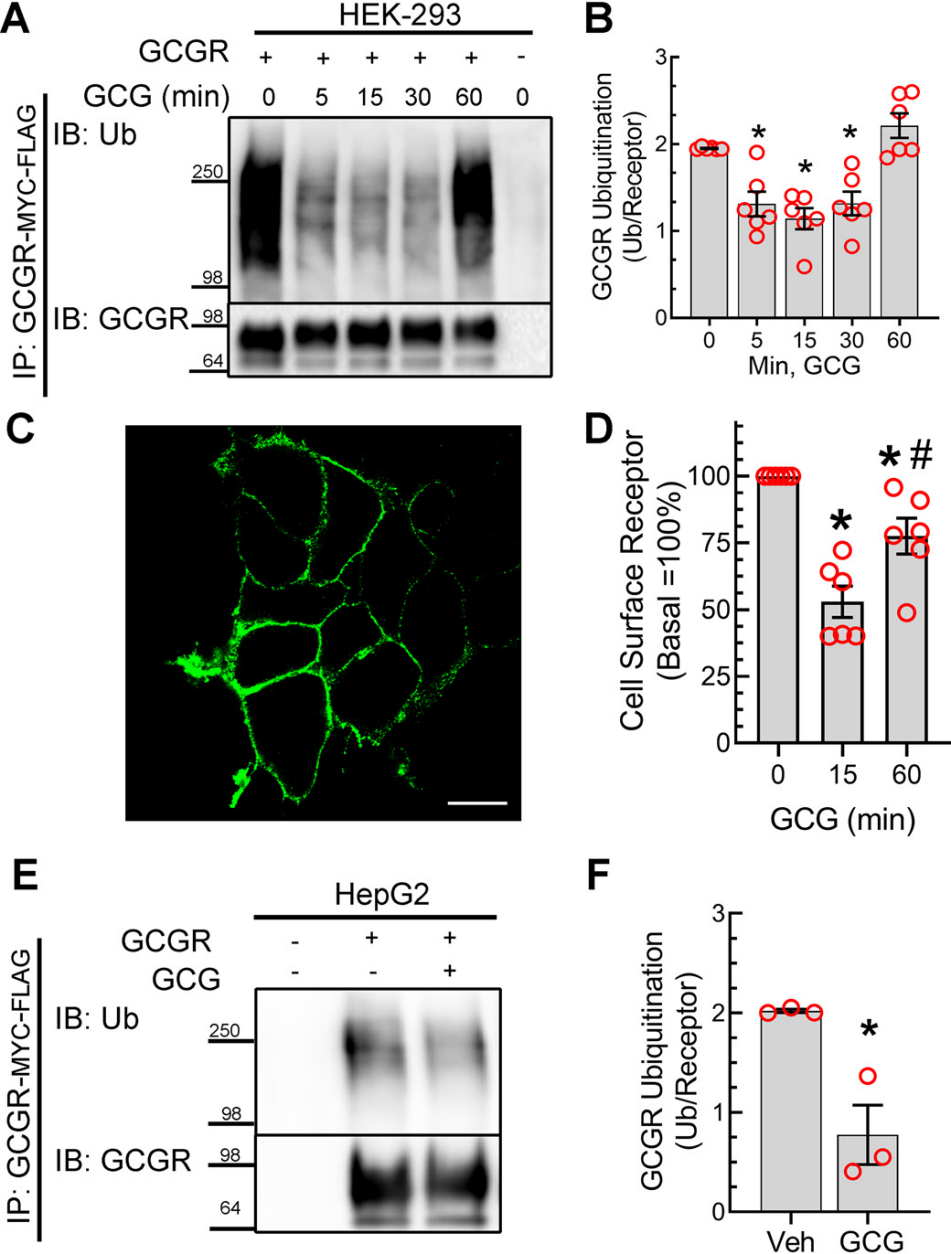
**GCG stimulation induces deubiquitination of the GCGR.***A*, HEK-293 cells stably expressing GCGR-MYC-FLAG were serum-starved for 1 h and then stimulated with 200 nm GCG for the indicated times. The receptor was immunoprecipitated with M2 anti-FLAG affinity gel (Sigma–Aldrich). GCGR ubiquitination was detected using the anti-ubiquitin antibody, FK1 (Enzo Life Sciences). The blot was then reprobed with an antibody that detects the MYC tag (SantaCruz Biotechnology Inc). *B*, the ubiquitinated smear was quantitated and normalized to cognate receptor bands and plotted as a ratio. The scatter graph represents the means ± S.E. (*error bars*) from six independent experiments. *, *p* < 0.05 *versus* control nonstimulated, one-way ANOVA, Bonferroni's multiple-comparison test. *C*, labeling of cell-surface MYC-GCGR with N-terminal MYC tag expressed in HEK-293 cells with anti-MYC IgG (Cell Signaling Technology) and Alexa 488–conjugated secondary antibody as visualized by a confocal microscope; *scale bar*, 10 μm. *D*, HEK-293 cells were transiently transfected with a plasmid encoding MYC-GCGR, and cell-surface receptor in nonpermeabilized cells was determined by an ELISA (see “Experimental procedures”). The scatter graph plotted as ±S.E. represents receptor expression on the cell surface with values from unstimulated cells taken as 100%. The data summarize measurements from two independent experiments, and each experiment is done in three independent sets of triplicates. *, *p* < 0.05 *versus* no GCG (0 min); #, *p* < 0.05 *versus* all others, one-way ANOVA and Bonferroni's multiple-comparison test. *E*, HepG2 cells stably expressing GCGR-MYC-FLAG were stimulated with 200 nm GCG for 15 min and further processed as in *A*. *F*, the ratio of ubiquitin smear over receptor expression plotted in the scatter graph is comprised of means ± S.E. from three independent experiments. *, *p* = 0.01 *versus* control nonstimulated, unpaired *t* test. *IP*, immunoprecipitation; *IB*, immunoblotting; *Ub*, ubiquitin; *Veh*, vehicle.

About 40 different GPCRs have been shown to be ubiquitinated, with a vast majority of these GPCRs undergoing ubiquitin-directed intracellular trafficking (20, 22). However, GCGR ubiquitination had not been characterized thus far. To define whether the GCGR is ubiquitinated and whether this is modulated by agonist stimulation, we treated human embryonic kidney (HEK-293) cells that have been stably transfected with GCGR with 200 nm glucagon for different times and determined the ubiquitination profile using FLAG immunoprecipitation by methods that we and others have reported before (14, 19, 23, 24). Interestingly, we not only observed an agonist-dependent mobility shift of the immunoprecipitated GCGR (see GCGR immunoblot in Fig. 1*A*) engendered by receptor phosphorylation (25, 26), but also detected an agonist-induced *decrease* in ubiquitination (*i.e.* deubiquitination) of the GCGR within 5 min, which was unchanged until 60 min of glucagon stimulation, when the GCGR ubiquitination returned to basal levels (Fig. 1, *A* and *B*). Within this duration of agonist exposure, we did not observe a significant change in total receptor protein, suggesting that the decrease in ubiquitination is not from a corresponding decrease in the GCGR expression. We also failed to detect GCGR ubiquitin signals with an antibody that is selective to monoubiquitin (P4D1), but antibodies that are selective to polyubiquitin (clone FK1 and rabbit polyclonal ubiquitin antibodies) yielded reproducible signals, which were detected only in receptor pulldowns and not in control immunoprecipitates; therefore, we conclude that the GCGR is polyubiquitinated (Fig. 1*A*).

To discern a correlation between glucagon-induced deubiquitination and internalization kinetics of the GCGR, we assessed agonist-induced changes in cell-surface expression of GCGR. We used a GCGR construct with an N-terminal MYC tag that could be detected at the plasma membrane in nonpermeabilized cells (Fig. 1*C*). We determined the amount of cell-surface GCGR in quiescent and agonist-stimulated cells using an ELISA (Fig. 1*D*). We mainly focused on 15 and 60 min of agonist stimulation, which showed distinct patterns of deubiquitination and reubiquitination (Fig. 1, *A* and *B*). At 15 min of glucagon stimulation, we detected a significant decrease in cell-surface GCGR expression compared with unstimulated conditions, and furthermore, after 60 min of glucagon stimulation, we observed a significant level of recovery of cell-surface receptors, although it did not attain levels in unstimulated cells (Fig. 1*D*). These data strongly suggest a correlation between GCGR deubiquitination and internalization at 15 min and GCGR reubiquitination and cell-surface recovery at 60 min.

The GCGR is expressed at higher levels in the liver than in other organs like kidney or pancreatic islets; hence, we also tested GCGR ubiquitination in the commonly used hepatocyte model cell line, HepG2. Glucagon-induced deubiquitination of the GCGR observed in HEK-293 cells is recapitulated in the HepG2 cells (Fig. 1, *E* and *F*) as we detected basal polyubiquitination of the GCGR, which decreased by ∼65% after 15 min of glucagon exposure. Thus, the GCGR presents a unique pattern of agonist-induced deubiquitination that is not observed with other GPCRs that have been tested for ubiquitination (16, 20).

### Glucagon-induced deubiquitination of the GCGR is blocked by sucrose, Dyngo-4a, and monensin

**Figure 2:**
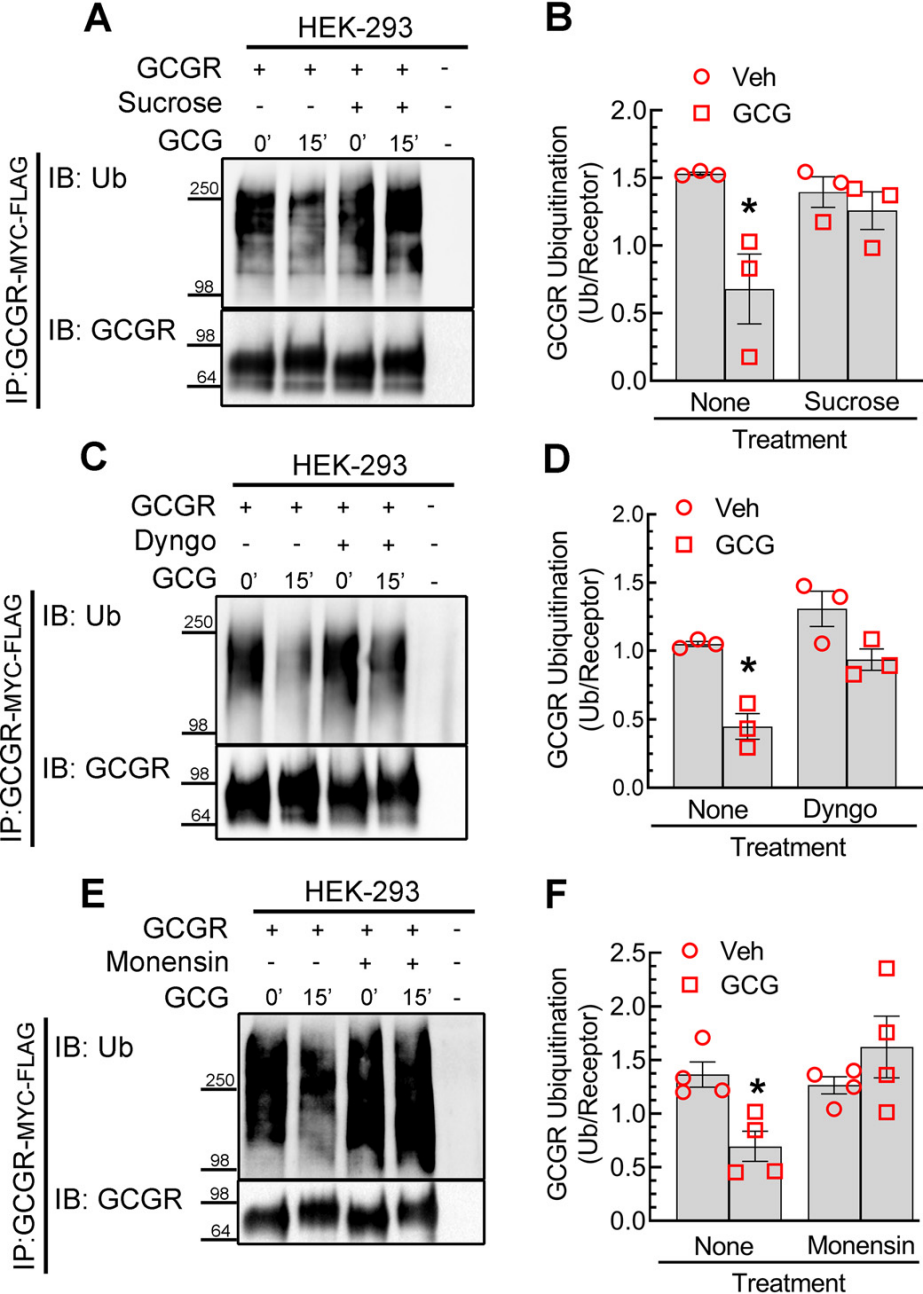
**Glucagon-induced deubiquitination of the GCGR is blocked by sucrose, Dyngo-4a, and monensin.** HEK-293 cells with stable expression of GCGR-MYC-FLAG were processed as in Fig. 1, except that cells were preincubated with sucrose (0.4 m) for 15 min (*A*), Dyngo-4a (60 μm) for 15 min (*C*), or monensin (50 μm) (*E*) for 1 h before agonist stimulation. The ubiquitin smear in each lane was normalized to the respective GCGR band and plotted as ratios for sucrose (*B*), Dyngo-4a (*D*), and monensin (*F*). Each scatter plot with *bars* summarizes means ± S.E. (*error bars*) from three (*B* and *D*) or four (*F*) independent experiments. *, *p* < 0.05 compared with vehicle and no treatment, two-way ANOVA, Holm–Sidak's multiple-comparison test. *IP*, immunoprecipitation; *IB*, immunoblotting; *Ub*, ubiquitin; *Veh*, vehicle.

Based on the correlation between glucagon-induced deubiquitination and internalization (Fig. 1), we hypothesized that the plasma membrane–localized receptors represent the ubiquitinated species and that internalized receptors are deubiquitinated units. If this were true, then inhibition of internalization should prevent glucagon-induced deubiquitination of the GCGR. The GCGR has been reported to utilize multiple pathways of internalization invoking both clathrin and caveolin-dependent mechanisms (8, 27). When we pretreated GCGR-stable cells with 0.4 m sucrose, which blocks clathrin-dependent and non-clathrin-dependent endocytosis (28), the agonist-induced deubiquitination of the GCGR was completely blocked (Fig. 2, *A* and *B*), suggesting that only internalized GCGRs are deubiquitinated. To assess the contribution of dynamin and clathrin-dependent endocytosis, we employed Dyngo-4a, a widely used chemical inhibitor of dynamin GTPase (29, 30, 31). When cells were pretreated with 60 μm Dyngo-4a, we detected a modest increase in basal ubiquitination, and although we observed a glucagon-induced decrease in ubiquitination, it was not significant (Fig. 2, *C* and *D*). Thus, blocking clathrin-dependent internalization prevented deubiquitination of most but perhaps not all activated GCGRs.

We next tested whether internalized GCGR is deubiquitinated when intracellular trafficking of the GCGR is disrupted. We used the carboxylic ionophore monensin, which has no effect on receptor internalization but blocks trafficking of internalized receptor complexes into recycling vesicles. These blocking effects of monensin on trafficking have been documented for several GPCRs as well as for other cell-surface receptors (32, 33, 34, 35). When GCGR-stable cells were pretreated for 60 min with 50 μm monensin, basal ubiquitination of the GCGR was unaltered compared with cells that were not treated with monensin. However, glucagon stimulation did not induce GCGR deubiquitination in just monensin-treated cells (Fig. 2, *E* and *F*). These results suggest that agonist-activated GCGRs have to be mobilized into specific compartments after internalization to be deubiquitinated.

### Reciprocal effects of monensin on GCGR trafficking into early versus recycling endosomes

**Figure 3:**
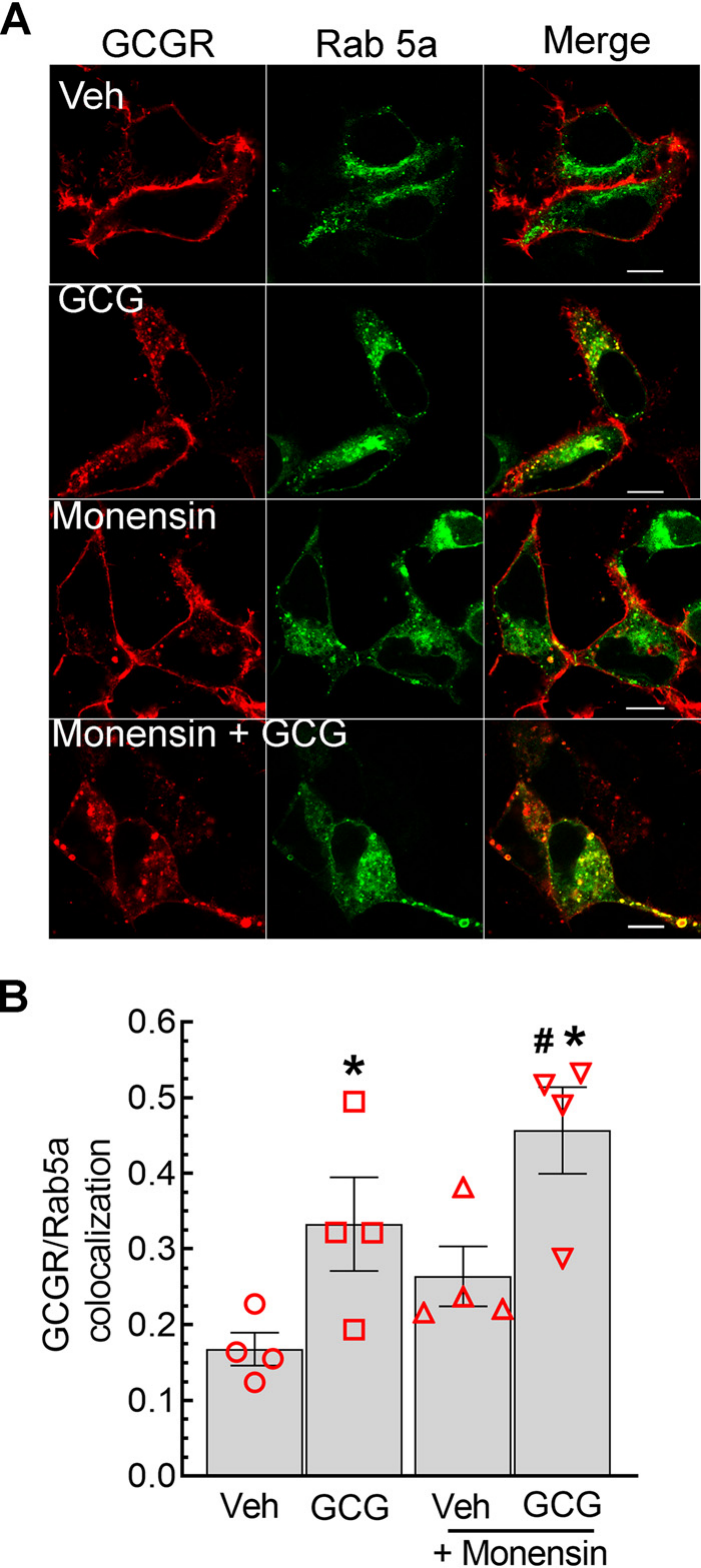
**Monensin increases the colocalization of GCGR and Rab5a.***A*, HEK-293-GCGR-MYC-FLAG cells transiently transfected with GFP-Rab5a were serum-starved for 1 h, preincubated with vehicle or 50 μm monensin for 60 min, and then stimulated with 1 μm GCG for 15 min. Cells were fixed, permeabilized, and immunostained for GCGR, using anti-MYC (clone 9E-10, Santa Cruz Biotechnology) primary antibody followed by Alexa Fluor 594–conjugated anti-mouse secondary antibody. Confocal images depict GCGR in *red* (Alexa 594) and Rab5a in *green*. Confocal images shown are representative of four independent experiments and ≥20 images for each condition. *B*, the scatter plot with *bars* summarizes means ± S.E. (*error bars*) of Pearson's correlation coefficients for GCGR and Rab5a colocalization determined using ImageJ software. *, *p* < 0.05 *versus* vehicle only; #, *p* < 0.05 *versus* GCG only, two-way ANOVA and Holm–Sidak's multiple-comparison test. *Scale bar*, 10 μm. *Veh*, vehicle.

Monensin can block GPCR recycling from different populations of endosomal vesicles, including early and perinuclear endosomes (36). Therefore, we next determined the effects of monensin on the subcellular localization of GCGR after 15 min of agonist activation that obtains the maximal extent of deubiquitination (Fig. 1). We first tested colocalization of GCGR and GFP-Rab5a, a small GTPase that is a well-known marker for the early endosome population (37, 38). As shown in the confocal images in Fig. 3*A*, in unstimulated GCGR stable cells, the staining for the GCGR was mostly detected at the plasma membrane, and GFP-Rab5a was mostly distributed in cytoplasmic vesicles. In cells stimulated with glucagon, we detected a significant increase in the colocalization of GCGR and GFP-Rab5a (Fig. 3, *A* and *B*). Monensin treatment alone led to a modest increase in GCGR localization in vesicles that were Rab5a-positive, resulting in a 20–30% increase in GCGR and Rab5a colocalization compared with unstimulated vehicle-treated cells. Furthermore, in cells exposed to both monensin and glucagon, we observed a significant increase in the colocalization of GCGR with GFP-Rab5a (Fig. 3, *A* and *B*). Accordingly, monensin potentiates glucagon-induced localization of GCGR in Rab5a-positive early endosomes.

**Figure 4:**
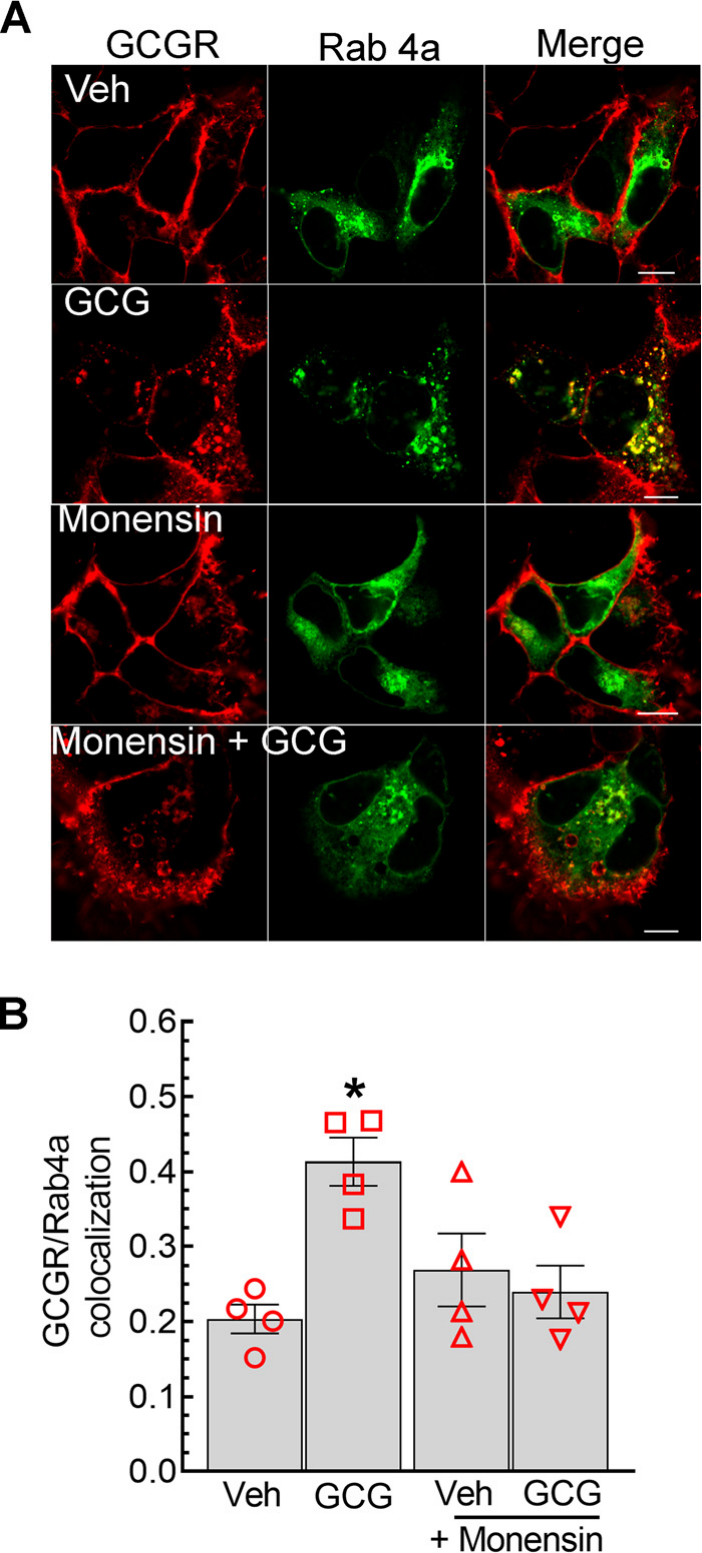
**Colocalization of GCGR and Rab4a is attenuated by monensin.***A*, HEK-293 cells stably transfected with GCGR-MYC-FLAG and transiently transfected with GFP-Rab4a, were serum-starved for 1 h, preincubated with vehicle or 50 μm monensin for 60 min, and then stimulated with 1 μm GCG for 15 min. Cells were processed for confocal imaging as in Fig. 3. Confocal images shown are representative of four independent experiments with ≥20 images acquired for each condition. *B*, the graphs plotted as means ± S.E. (*error bars*) represent Pearson's correlation coefficients for GCGR and Rab4a colocalization that were determined using ImageJ software. *, *p* < 0.05 *versus* the rest, two-way ANOVA and Holm-Sidak's multiple-comparison test. *Scale bar*, 10 μm. *Veh*, vehicle.

We next repeated the confocal experiments to determine trafficking of the GCGR with or without monensin with another Rab marker protein, GFP-Rab4a, which localizes predominantly at recycling endosomes. As shown in Fig. 4 (*A* and *B*), glucagon stimulation leads to a robust increase in GCGR and GFP-Rab4a colocalization compared with unstimulated cells. On the other hand, in a majority of monensin-treated cells, glucagon stimulation caused GCGR to prevail in large vesicles that did not colocalize with GFP-Rab4a. Accordingly, monensin prevents the trafficking of internalized GCGRs into Rab4a-marked recycling vesicles. Taken together with the effects on GCGR ubiquitination (Fig. 2), these data suggest that in monensin-treated cells, ubiquitinated GCGRs are accumulated in Rab5a-positive early endosomes.

### Reciprocal effects of dominant negative Rab4a and Rab5a on GCGR deubiquitination

**Figure 5:**
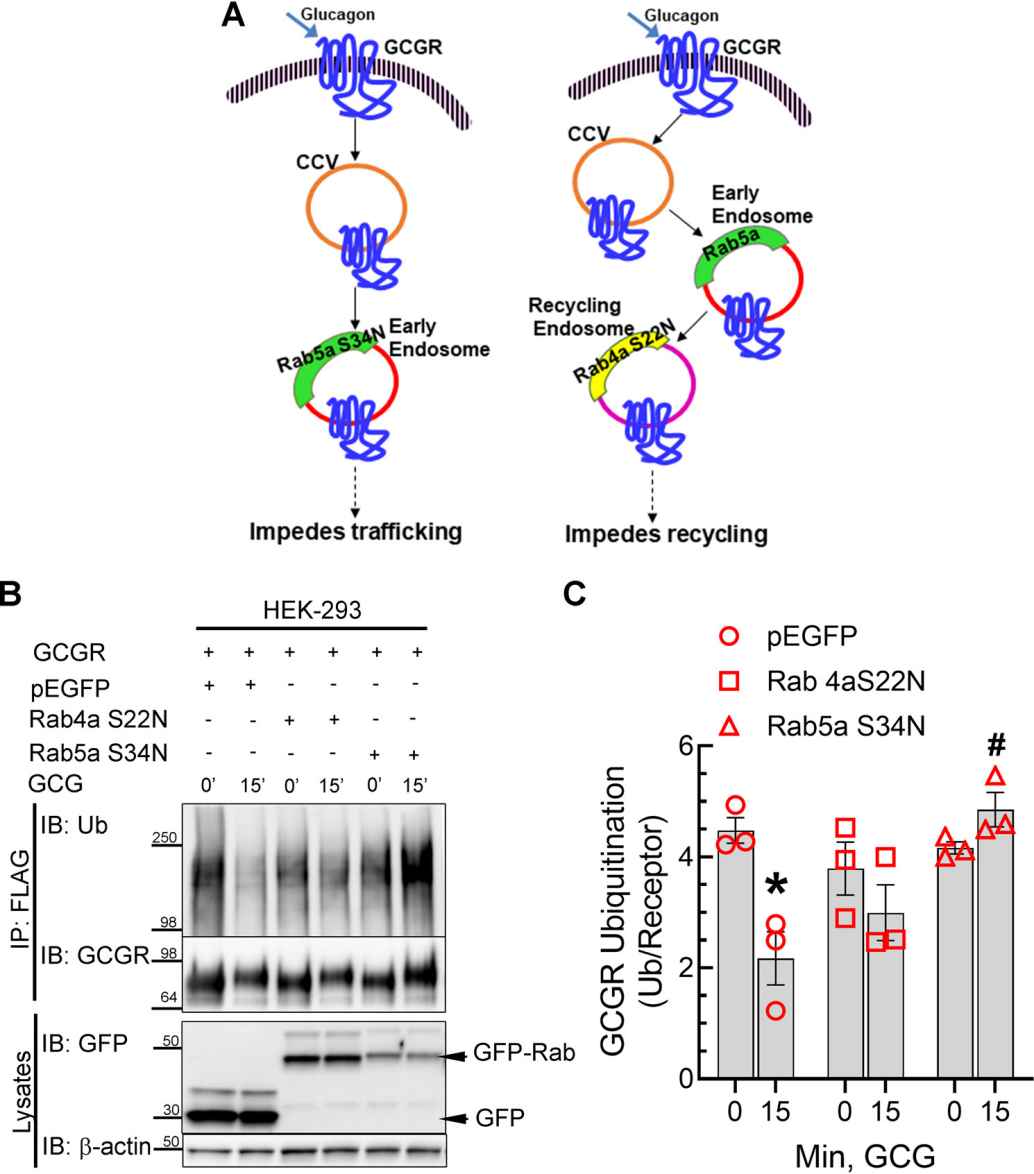
**Reciprocal effects of dominant negative Rab4a and Rab5a on GCGR deubiquitination.***A*, schematic showing effects of dominant negative Rab4aS22N and Rab5aS34N on endocytic and recycling pathways. *B*, HEK 293 cells stably expressing GCGR-MYC-FLAG were transfected transiently with GFP, GFP-Rab4aS22N, and GFP-Rab5aS34N (dominant negative) mutants. Cells were stimulated with 200 nm GCG for 15 min and subjected to FLAG immunoprecipitation. The immunoprecipitates and whole-cell lysates were resolved by SDS-PAGE and immunoblotted sequentially for the indicated proteins. Ubiquitin smears were quantitated and normalized to their cognate GCGR-MYC-FLAG bands and plotted as a ratio of ubiquitin over receptor. The graphs include means ± S.E. (*error bars*) from three independent experiments. *C*, *, *p* < 0.05 *versus* no GCG (0 min); #, *p* < 0.05 *versus* rest of the 15-min GCG, two-way ANOVA and Holm–Sidak's multiple comparison test. *IP*, immunoprecipitation; *IB*, immunoblotting; *Ub*, ubiquitin.

To further dissect the contrasting effects produced by monensin on the colocalization of the internalized GCGR with Rab4a and Rab5a, we next employed the inactive (dominant negative) mutants in our ubiquitination assays. Whereas the WT Rab GTPases bind GTP and trigger GTP hydrolysis and endosomal vesicle fusion, Rab4a S22N and Rab5a S34N mutants have increased affinity for GDP and are unable to engender vesicle membrane fusion (39, 40). These mutants act as dominant negatives by sequestering endogenous guanine nucleotide exchange factors because of the low affinity for GTP (41). Exogenous expression of Rab4a S22N blocks recycling of membrane receptors to the plasma membrane, and Rab5a S34N has been shown to either inhibit internalization and retain receptors at the plasma membrane or trap receptors in small endocytic vesicles that are destined to fuse with early endosomes (Fig. 5*A*). Co-expression of GFP vector preserved basal ubiquitination of GCGRs as well as agonist-induced deubiquitination of the GCGR (Fig. 5, *B* and *C*). In cells transfected with GFP-Rab4a S22N, basal GCGR ubiquitination was modestly reduced compared with GFP-transfected cells, and agonist stimulation led to further deubiquitination. In marked contrast, in cells transfected with GFP-Rab5a S34N, we detected a 25% increase in ubiquitination after agonist activation, compared with unstimulated samples. We also assessed the effect of GFP-Rab5a S34N on GCGR subcellular distribution by confocal microscopy. As shown in Fig. S1, in cells expressing GFP-Rab5a S34N, we detected GCGR internalization into small vesicles that did not colocalize with GFP-Rab5a S34N. On the other hand, in cells expressing GFP-Rab5a, WT construct, we detected a robust colocalization of internalized GCGRs with GFP-Rab5a. Thus, only when we blocked GCGR trafficking before its entry into Rab5a-marked early endosomes did we prevent GCGR deubiquitination. Moreover, blocking GCGR exit trafficking from recycling endosomes with Rab4a S22N still enabled GCGR deubiquitination. Accordingly, we infer that internalized GCGRs are deubiquitinated at Rab5a early endosomes and then sort into Rab4a recycling endosomes.

### Endocytic recycling of the GCGR is choreographed by two distinct deubiquitinases

To gain further insights into the molecular connection between GCGR deubiquitination and trafficking, we next screened for the deubiquitinating enzyme involved in GCGR trafficking. About 85 functional deubiquitinases (DUBs) are expressed in human cells and are classified into five subfamilies based on the catalytic domain and mechanisms involved (42). The ubiquitin-specific protease subfamily contains ∼50 DUBs, and several of them have been associated with GPCR regulation. We undertook an approach of testing candidate DUBs based on their unique catalytic mechanism enabling deubiquitination and ubiquitination (A20) (43, 44), subcellular localization at early endosomes (USP10 and STAMBP) (42), and their established roles in GPCR trafficking (USP14, USP20, and USP33) (18, 45).

Because the GCGR presents a unique pattern of basal ubiquitination and agonist-induced deubiquitination and subsequent reubiquitination (Fig. 1), we suspected that a DUB with ubiquitin-editing properties such as A20 might be a cognate DUB for the GCGR. A20, which mitigates apoptotic death of pancreatic β cells, is a unique DUB, possessing both deubiquitinase and E3 ubiquitin ligase activities in a single protein unit (43, 44). A20 can deubiquitinate substrates and concomitantly append new ubiquitin moieties; thus, its actions will favor the rapid deubiquitination and reubiquitination of the GCGR induced by glucagon (Fig. 1). To establish the association of A20 and the GCGR, we immunoprecipitated the GCGR-MYC-FLAG with anti-FLAG affinity beads from the stable cells and immunoblotted the eluates for endogenously expressed A20 in HEK-293 cells. A FLAG immunoprecipitate from HEK-293 cells transfected with vector served as our negative control to define specific association of A20. Contrary to our expectation, agonist stimulation decreased the interaction of A20 and GCGR (Fig. S2), suggesting that A20 is unlikely to serve as a cognate DUB for the GCGR during endocytic recycling.

USP10, which is detected in nuclear and endosomal locations, has been linked with endocytic recycling of the cystic fibrosis transmembrane conductance regulator and has been shown to deubiquitinate and stabilize sorting nexin 3 that regulates recycling (46, 47). However, our co-immunoprecipitation assays did not reveal a significant increase in the association between USP10 and GCGR (Fig. S3, A and B). Concordantly, siRNA knockdown of USP10, which caused a down-regulation of USP10 expression by >80%, did not prevent agonist-induced deubiquitination of the GCGR (Fig. S3, C and D). Thus, USP10, despite its known activity at sorting endosomes, does not act as a deubiquitinase for the GCGR.

**Figure 6:**
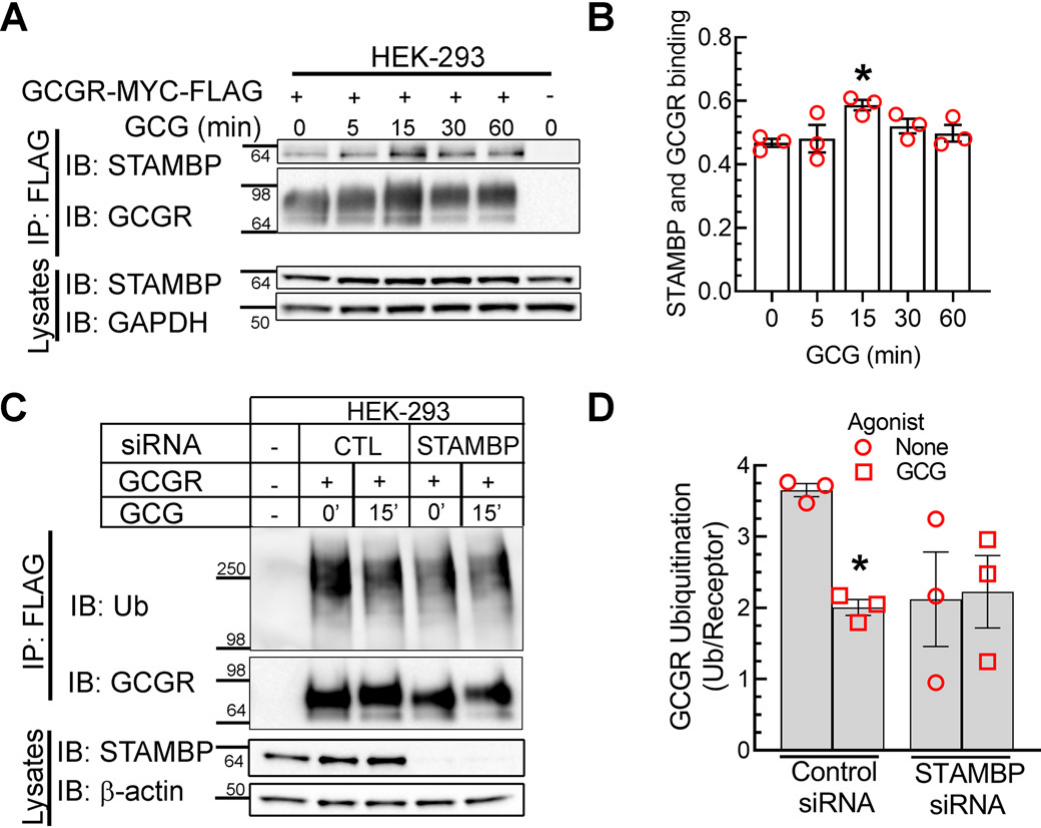
**STAMBP interacts with and deubiquitinates the GCGR.***A*, HEK-293 cells stably expressing GCGR-MYC-FLAG receptor were serum-starved for 1 h and stimulated with 200 nm GCG for the indicated times followed by immunoprecipitation with anti-FLAG agarose beads. The immunoprecipitates were resolved on 4–20% Tris-glycine gels and immunoblotted for the indicated proteins. *B*, scatter plot with *bars* summarizes quantification of STAMBP normalized to receptor level from three independent experiments. *, *p* < 0.05 *versus* unstimulated (0 min) condition, one-way ANOVA, Bonferroni's multiple-comparison test. *C*, HEK-293 cells stably expressing GCGR-MYC-FLAG were transfected with nontargeting siRNA (control) or siRNA targeting STAMBP. Cells were stimulated with 200 nm GCG, followed by receptor immunoprecipitation and immunoblotting for ubiquitin. *B*, the ubiquitinated smear was normalized to the cognate receptor band and plotted as means ± S.E. (*error bars*) from three independent experiments. *, *p* ˂ 0.05 compared with control siRNA, nonstimulated conditions, two-way ANOVA, Holm–Sidak's multiple-comparison test. *IP*, immunoprecipitation; *IB*, immunoblotting; *Ub*, ubiquitin.

STAMBP is a metalloprotease and a deubiquitinase classified under the JAMM (JAB1/MPN/Mov34) subfamily of DUBs and associates with clathrin on budding endosomes (48, 49). STAMBP has been shown to regulate endocytic sorting of the GPCRs CXCR4 and PAR2 (50, 51) by engaging ESCRT proteins. We therefore assessed whether STAMBP can bind the GCGR and whether STAMBP knockdown alters GCGR ubiquitination status. As shown in Fig. 6 (*A* and *B*), STAMBP associates with the GCGR, and the interaction is significantly increased after agonist stimulation for 15 min. Additionally, siRNA-mediated knockdown of STAMBP drastically reduces basal ubiquitination of GCGR, and no further deubiquitination ensues with glucagon stimulation (Fig. 6, *C* and *D*). When a cognate DUB is depleted, it is expected that substrate ubiquitination will increase; hence, the absence of such an effect on the GCGR with STAMBP knockdown raises questions about this DUB's role in GCGR regulation. On the other hand, although GCGR ubiquitination signals are reduced under basal conditions with STAMBP knockdown, glucagon stimulation does not induce further deubiquitination in cells with STAMBP knockdown (Fig. 6, *C* and *D*). Accordingly, STAMBP might exclusively deubiquitinate activated, internalized GCGR, without having any effect on the basal ubiquitination of plasma membrane–localized GCGR. Additionally, in the absence of STAMBP, hyperactivity of another DUB can lead to enhanced deubiquitination of the GCGR under basal conditions. Overall, these paradoxical findings suggest that the regulation of GCGR is complex and involves more than one DUB during endocytic recycling.

As a targeted approach to identify the cognate DUBs for the GCGR, we tested the effects of USP14, USP20, and USP33 that have been connected with GPCR trafficking. USP14 regulates lysosomal degradation of the CXCR4 and metabotropic glutamate receptor (45, 52). However, USP14 knockdown did not block glucagon induced deubiquitination of the GCGR (Fig. S4, A and B). USP20, which regulates β_1_- and β_2_-ARs (18, 19), also does not deubiquitinate GCGR, because in cells with USP20 knockdown, the extent of glucagon-induced deubiquitination is identical to cells with control knockdown (Fig. S4, C and D).

**Figure 7:**
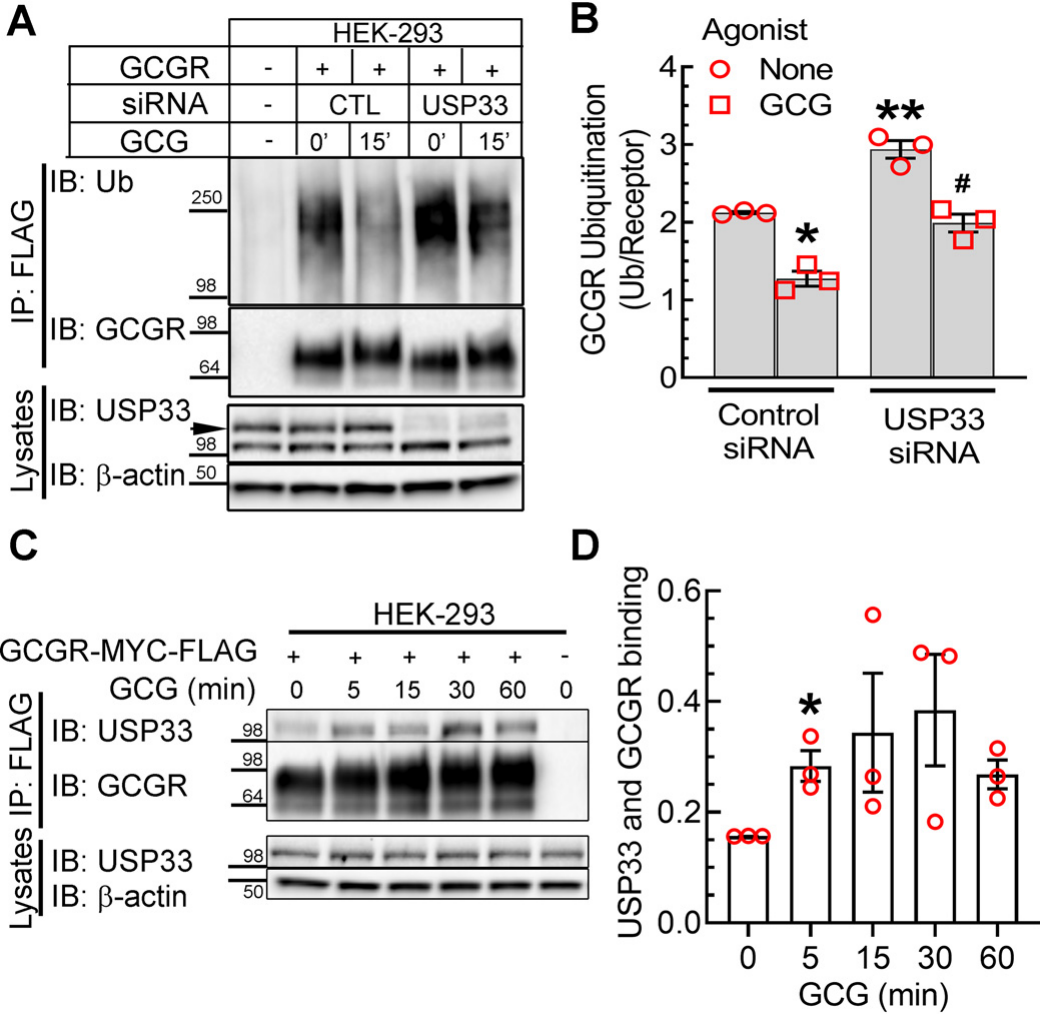
**USP33 deubiquitinates the GCGR in quiescent and agonist-treated cells.***A*, HEK-293 cells with stable expression of GCGR-MYC-FLAG were transfected with siRNA targeting no mRNA (control) or USP33 mRNA. Cells were serum-starved for 1 h and stimulated with 200 nm GCG for 15 min, followed by immunoprecipitation with anti-FLAG agarose gel. Subsequent steps were as described in Fig. 1. *B*, the graph represents means ± S.E. (*error bars*) from three independent experiments. *, *p* < 0.05 *versus* no agonist, control siRNA; **, *p* <0.05 *versus* the rest; #, *p* < 0.05 *versus* GCG, control siRNA, two-way ANOVA and Holm–Sidak's multiple-comparison test. *C*, HEK-293 cells stably expressing GCGR-MYC-FLAG receptor were serum-starved for 1 h and stimulated with 200 nm GCG for the indicated times followed by immunoprecipitation with anti-FLAG agarose beads. The immunoprecipitates were resolved on 4–20% Tris-glycine gels and immunoblotted for the indicated proteins. *D*, scatter plot with *bars* summarizes quantification of USP33 normalized to receptor level from three independent experiments. *, *p* < 0.05 *versus* unstimulated (0 min) condition, one-way ANOVA, Bonferroni's multiple-comparison test. *IP*, immunoprecipitation; *IB*, immunoblotting; *Ub*, ubiquitin.

In contrast to the lack of any impact of USP14 and USP20, when we knocked down USP33 in the GCGR stable cells, we observed a 40–50% *increase* in basal ubiquitination of GCGR (Fig. 7, *A* and *B*). However, glucagon stimulation of USP33-depleted cells still caused a reduction in GCGR ubiquitination compared with unstimulated cells. Nonetheless, comparing just the agonist-stimulated conditions, the amount of ubiquitinated GCGRs was significantly higher in USP33-depleted cells than in control cells (Fig. 7, *A* and *B*). Co-immunoprecipitation assays showed a correlating binding pattern of USP33 and the GCGR. We could detect specific binding under nonstimulated conditions, and this interaction increased with agonist activation, demonstrating a significant increase at 5 min of agonist treatment. The USP33-GCGR complex likely dissembles at later times of agonist, as suggested by the decreasing interaction seen in the pulldown assay at 60 min of agonist stimulation (Fig. 7, *C* and *D*).

We also tested a catalytically inactive USP33 in which the cysteine and histidine residues of the active site are mutated (USP33-CH) for its effect on GCGR ubiquitination. Previous studies have indicated that this mutant behaves as a dominant negative and can compete with endogenous USP33 (18). When we overexpressed USP33-CH in GCGR stable HEK-293 cells, we observed a significant increase in basal ubiquitination of the GCGR, but the mutant construct did not have a similar pronounced effect in blocking deubiquitination of agonist-activated GCGRs (Fig. S5, A and B). These results strongly support the notion that USP33 is a cognate DUB for the GCGR and limits the level of ubiquitinated GCGRs in a constitutive manner.

**Figure 8:**
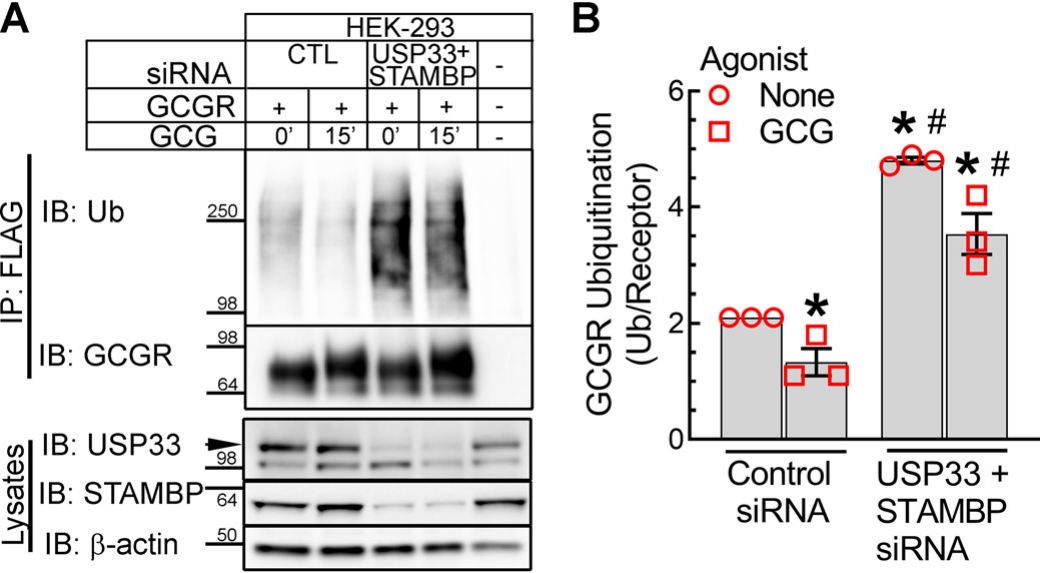
**GCGR is deubiquitinated by USP33 and STAMBP.***A*, HEK-293 cells stably expressing GCGR-MYC-FLAG were transfected with siRNA targeting either no mRNA or with siRNA mixture targeting UPS33 and STAMBP for 48 h and stimulated with 200 nm GCG for 15 min. Immunoprecipitates and cell lysates were immunoblotted for the indicated proteins. *B*, quantification of ubiquitination normalized to respective GCGR bands shown as means ± S.E. (*error bars*) from three independent experiments. *, *p* < 0.05 *versus* control, no agonist; #, *p* < 0.05 *versus* control, GCG, two-way ANOVA and Holm–Sidak's multiple-comparison test. *IP*, immunoprecipitation; *IB*, immunoblotting; *Ub*, ubiquitin.

Our experiments also suggest that USP33 deubiquitinates agonist-activated GCGRs, but its role is shared with another DUB, which is perhaps STAMBP. Therefore, we next determined the effects of simultaneously knocking down both USP33 and STAMBP on GCGR deubiquitination (Fig. 8, *A* and *B*). This double knockdown produced a dramatic increase in GCGR ubiquitination in both unstimulated and glucagon-stimulated cells compared with cells with control knockdown (Fig. 8, *A* and *B*). As per the quantitation summary included in Fig. 8*B*, we still obtained a modest decrease in ubiquitination after glucagon stimulation in cells with double knockdown, which was not statistically significant compared with GCGR ubiquitination in the corresponding unstimulated condition. We attribute this amount of agonist-induced deubiquitination to insufficient concurrent knockdown of both DUBs in the GCGR stable cells. Overall, these data strongly suggest that both STAMBP and USP33 deubiquitinate agonist-activated GCGR, whereas the basal ubiquitination of GCGR is governed only by USP33 activity.

### A mutant GCGR impaired in ubiquitination is augmented in agonist-induced recycling

**Figure 9:**
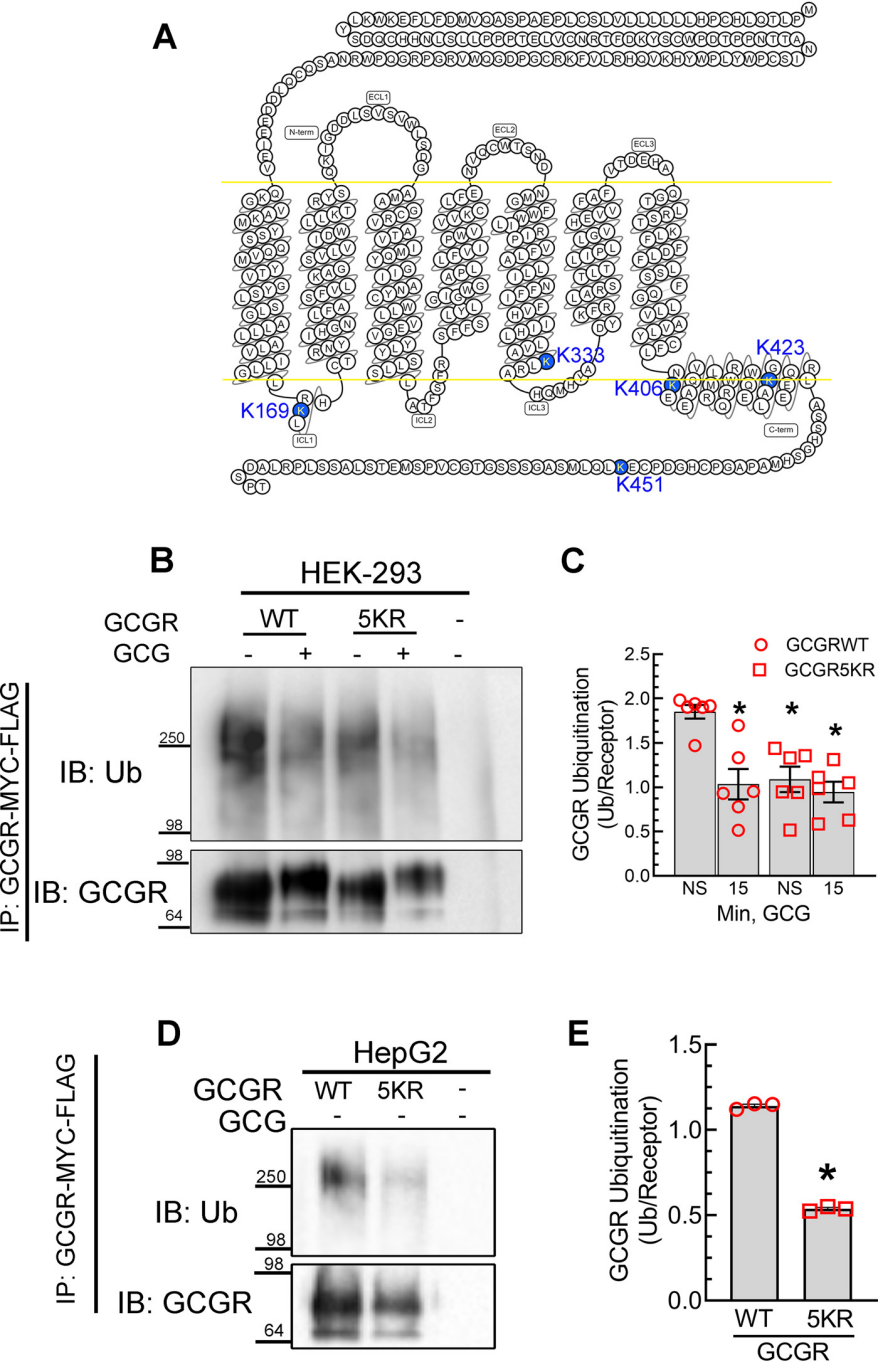
**GCGR with intracellular lysines mutated to arginines is impaired in ubiquitination.***A*, snake plot of the GCGR, indicating positions of lysine residues (highlighted in *blue*) that are mutated to arginine residues in GCGR-5KR. Snake plot was obtained from gpcrdb.org. *B*, HEK-293 cells stably expressing either GCGR-MYC-FLAG or GCGR-5KR-MYC-FLAG were stimulated with 200 nm GCG for 15 min. The receptor was immunoprecipitated using FLAG-affinity gel, followed by serial immunoblotting for ubiquitin and GCGR as in Fig. 1. *C*, the graph summarizes means ± S.E. (*error bars*) from six independent experiments. *, *p* < 0.05 *versus* GCGR WT, unstimulated conditions, two-way ANOVA and Holm–Sidak's multiple-comparison test. *D*, HepG2 cells stably expressing GCGR-MYC-FLAG or GCGR-5KR-MYC-FLAG were analyzed for ubiquitination as in Fig. 1. *E*, the graph summarizes means ± S.E. from three independent experiments. *, *p* < 0.001, unpaired *t* test. *IP*, immunoprecipitation; *IB*, immunoblotting; *Ub*, ubiquitin.

GPCR ubiquitination is generally targeted at lysine residues in the intracellular loops and/or the carboxyl tail (14, 15, 24, 53), although in a few cases, noncanonical amino acids (Cys and Ser) serve as sites of ubiquitination (54, 55). Previous studies have indicated redundancy in ubiquitination sites of GPCRs, which is also true for many substrates that have multiple lysine residues (24, 56, 57). We therefore replaced all five lysines in the intracellular domains (Fig. 9*A*) with arginine residues (Lys to Arg mutation preserves the charge but averts ubiquitination) to generate GCGR-5KR. This mutant expressed at comparable levels as the WT GPCR in HEK-293 cells, but its basal ubiquitination was significantly reduced, and glucagon stimulation had little effect in reducing the ubiquitination signal (Fig. 9, *B* and *C*). Accordingly, the lysines in the intracellular loops of GCGR are the critical sites for ubiquitination. We also compared the basal ubiquitination of GCGR WT and GCGR-5KR in HepG2 cells. As shown in Fig. 9 (*D* and *E*), the lysine mutant is significantly impaired in basal ubiquitination. The remnant ubiquitin signals detected in the GCGR-5KR could be from ubiquitination at nonlysine residues; however, this ubiquitination is unaffected by glucagon stimulation (Fig. 9, *B* and *C*).

**Figure 10:**
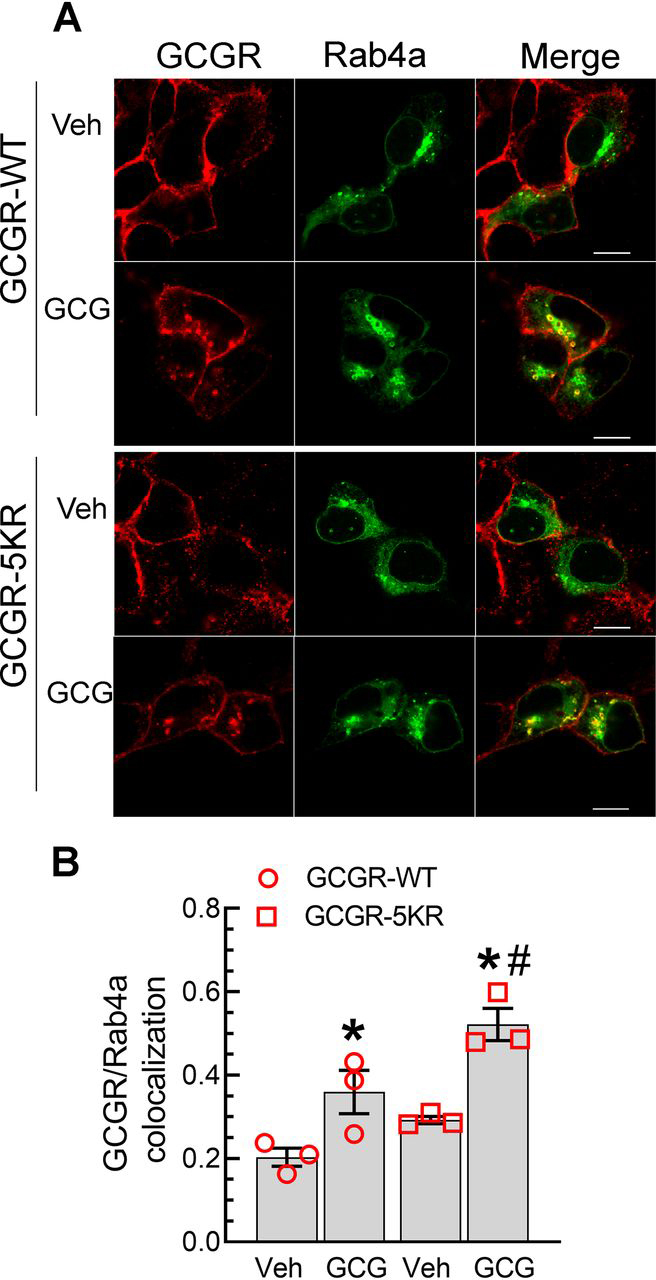
**Obliteration of ubiquitination sites in the GCGR augments its agonist-induced recycling.** HEK-293 cells stably expressing GCGR or GCGR-5KR were transfected transiently with GFP-tagged Rab4a (*A*) serum-starved for 60 min and stimulated with 1 μm GCG for 15 min. The colocalization of the GCR and GFP-Rab4a was determined as in Fig. 4. Images shown are from one of three independent experiments and ≥19 images for each condition. *B*, quantification of colocalization shown as Pearson's correlation co-efficient; *, *p* < 0.05 *versus* GCGR-WT vehicle; #, *p* < 0.05 *versus* GCGR-WT GCG, two-way ANOVA and Holm–Sidak's test. *Scale bar*, 10 μm. *Error bars*, S.E. *Veh*, vehicle.

Based on our analyses with monensin (Figure 2, Figure 3, Figure 4), if the GCGR is preserved in a ubiquitinated state, its trafficking into Rab4a recycling endosomes is ablated. To address whether a reduction in GCGR ubiquitination would affect the endocytic recycling of GCGR, we compared the colocalization of GFP-Rab4a with WT and 5KR GCGRs (Fig. 10, *A* and *B*). We observed a significant increase in the colocalization of internalized GCGR-5KR with Rab4a after glucagon stimulation, compared with the colocalization between GCGR WT and Rab4a (Fig. 10, *A* and *B*). Additionally, the mutant GCGR, which is impaired in ubiquitination colocalized increasingly with Rab4a even under basal conditions. Thus, the deubiquitinated state of the GCGR facilitates endocytic recycling through Rab4a vesicles.

## Discussion

Our findings strongly suggest that plasma membrane–localized GCGR is ubiquitinated on intracellular lysine residue(s) and that glucagon stimulation induces rapid internalization and concomitant deubiquitination of the internalized GCGR. We also demonstrate that deubiquitination of the GCGR occurs at Rab5a endosomes and involves two dissimilar DUBs, namely USP33 and STAMBP. Whereas USP33 deubiquitinates both unactive and agonist-bound GCGR, STAMBP deubiquitinates primarily agonist-activated and internalized GCGR at the Rab5a early endosome.

Although about 40 GPCRs have been characterized for the role of ubiquitination and many of them have been linked with an E3 ubiquitin ligase activity, only a limited number of studies have addressed the role of deubiquitinases in GPCR trafficking (16, 20). Our findings that glucagon rapidly induces deubiquitination of the GCGR likely represent a unique example. Our studies also identified that two highly dissimilar DUBs regulate GCGR endocytic recycling. USP33 belongs to the ubiquitin-specific protease subfamily, and its enzymatic activity relies on conserved Cys and His residues in the catalytic domain (58, 59). USP33 is localized in the cytoplasm, but its expression is enriched in endoplasmic reticulum, endosomes, and Golgi (18, 42). USP33 deubiquitinates the β_2_-AR and promotes β_2_-AR recycling, but USP33 does not deubiquitinate the β_1_-AR; additional substrates identified for USP33 include β-arrestin 2 and TRAF6, among other nonreceptor proteins (13, 18, 19, 60, 61, 62). USP33 knockdown drastically increases GCGR ubiquitination in quiescent cells; one possibility is that USP33 deubiquitinates and protects newly synthesized receptors from 26S proteasomal degradation. Whether USP33 regulates export trafficking of the GCGR and whether this involves deubiquitination at specific domains in the GCGR are important questions that need to be addressed in the future.

Unlike USP33, which is a cysteine protease, STAMBP (also called associated molecule with a Src homology 3 domain of signal transducing adaptor molecule, or AMSH) is a metalloprotease that mainly localizes at early endosomes. STAMBP has a Zn^2+^ molecule at the active site coordinated by two His, one Asp, and one Glu bridged by a catalytic water molecule (63). STAMBP has been linked with the trafficking of several receptors: epidermal growth factor receptor, calcium-sensing receptor, δ-opioid receptor, PAR2, and CXCR4 (48, 50, 51, 64). For some internalized receptors, the activity of STAMBP is linked with recycling, whereas for other receptors, STAMBP-mediated deubiquitination promotes receptors to be moved into multivesicular bodies and subsequently into lysosomes for degradation. STAMBP's regulation of endocytic machinery proteins, STAM and Grb2, directs trafficking of the CXCR4 into lysosomes (51). STAMBP-mediated deubiquitination is required for the early to late endosomal trafficking of PAR2 (50). In contrast, STAMBP-mediated deubiquitination appears to be critical for the trafficking of the GCGR into Rab4a recycling endosomes. Furthermore, we did not detect a robust colocalization of the GCGR and lysosomal marker protein LAMP2 unless cells were exposed to glucagon for longer times (4–6 h; data not shown). Therefore, deubiquitination of the GCGR by STAMBP after acute exposure to glucagon may not critically influence GCGR trafficking to the lysosomes. Future studies with prolonged agonist stimulation and comparison with other GPCRs such as the PAR2 may reveal mechanistic aspects of STAMBP activity and its differential regulation of lysosomal trafficking *versus* endocytic recycling of internalized GPCRs.

Our experiments with chemical inhibitors, sucrose, Dyngo-4a, and monensin, and the co-expression of Rab GTPases revealed interesting spatial and temporal correlation between GCGR trafficking and ubiquitination status. Rab GTPases constitute the largest family of small GTPases, and they are not only employed as marker proteins of intracellular compartments, but their activity defines vesicle fusion events and this can impact endocytic trafficking of GPCRs (38, 65). Rab proteins cycle between GDP-bound inactive and GTP-bound active conformations, and this correlates with their subcellular distributions: only GTP-bound form associates with membranes. Certain GPCRs can specifically bind Rab GTPases and influence their GTPase activity, as demonstrated for Rab5a by the angiotensin 1a receptor and for Rab11a by the β_2_-AR (66, 67, 68).

The deubiquitinated GCGR localizes in Rab4a vesicles within 15 min of agonist-stimulation, suggesting that most of the internalized GCGRs rapidly recycle to the plasma membrane. Our kinetic analyses also suggest that GCGRs are reubiquitinated with longer agonist-stimulation. Interestingly, E3 ligase activity at Rab4a endosomes has been reported (69), but our experiments with Rab4a DN, which trap GCGRs in recycling vesicles, did not accumulate ubiquitinated GCGRs, suggesting that the reubiquitination occurs after GCGRs exit Rab4a endosomes. Further extensive studies are needed to understand how plasma membrane–localized and recycled GCGRs are ubiquitinated. The GCGR-5KR, which is impaired in ubiquitination, does retain some ubiquitination signal that is unchanged with agonist stimulation. It is therefore tempting to speculate that the plasma membrane localization of GCGR may entail ubiquitination targeted at both lysine and nonlysine residues.

Our experiments revealed a characteristic gel mobility shift of agonist-activated GCGR, suggesting phosphorylation by GRKs (25, 26) and subsequent recruitment of β-arrestins. Interestingly, the mutant GCGR-5KR still presents this mobility shift, perhaps because GRK-mediated phosphorylation precedes internalization and deubiquitination events. Both β-arrestin 1 and β-arrestin 2 have been implicated in the internalization and recycling of GCGR in HEK-293 and hamster hepatocytes (8, 27). The exact role of each β-arrestin isoform in orchestrating agonist-induced deubiquitination and recycling of the GCGR remains to be defined.

Dysregulated GCGR trafficking has been linked with hyperglucagonemia and the development of pancreatic α-cell hyperplasia and neuroendocrine tumors. We propose that compounds that modulate the deubiquitinase activity of either USP33 or STAMBP may be of therapeutic use in the treatment of neuroendocrine tumors that result from impaired GCGR trafficking and signaling. In conclusion, we have shown that the glucagon receptor is basally ubiquitinated and is rapidly deubiquitinated with glucagon stimulation. The deubiquitination of internalized GCGRs is choreographed by two deubiquitinases, USP33 and STAMBP, to promote rapid recycling. Deciphering the role of ubiquitination and deubiquitination in regulating GCGR signal transduction and identifying the ubiquitination sites that connect signaling and trafficking of the GCGR are important questions for future research.

## Experimental procedures

### Reagents

Anti-FLAG M2 affinity agarose gel, *N*-ethylmaleimide, sucrose, monensin, Triton X-100, and BSA were purchased from Sigma. The following IgGs were procured from the sources listed: mouse monoclonal c-Myc (catalog no. SC-40) and rabbit polyclonal c-Myc (catalog no. SC-789) from Santa Cruz Biotechnology, Inc.; mouse monoclonal anti-β-actin (catalog no. A5441) from Sigma; anti-ubiquitin FK1 (BML-PW8805) from Enzo Life Sciences; rabbit polyclonal STAMBP (catalog no. 5245), rabbit monoclonal USP14 (catalog no. 11931), rabbit monoclonal USP10 (catalog no. 8501), rabbit monoclonal A20/TNFAIP3 (catalog no. 5630), rabbit polyclonal Myc tag (catalog no. 2272), rabbit monoclonal GAPDH (HRP conjugate, catalog no. 3683), and rabbit polyclonal ubiquitin (catalog no. 3933) antibodies from Cell Signaling Technology; and rabbit polyclonal anti-USP20 (A301-189A) and rabbit polyclonal anti-USP33 (A300-925A) from Bethyl Laboratories, Inc. Dyngo-4a® was purchased from Abcam. 1-Step^TM^ Turbo TMB-ELISA Substrate Solution was purchased from Thermo Scientific. HRP-conjugated secondary antibodies were from GE Biosciences, Cell Signaling Technology and Bethyl Laboratories, Inc. Lipofectamine 2000TM was purchased from Invitrogen. Alexa Fluor 594–conjugated secondary antibody was obtained from Invitrogen and used at a dilution of 1:500 for immunofluorescence labeling.

### Cell lines and plasmids

HEK-293 cells and the hepatocyte cell line HepG2 were obtained from the American Type Culture Collection and cultured in minimal essential medium supplemented with 10% fetal bovine serum and 1% penicillin/streptomycin. HepG2 cells were maintained in minimum essential medium supplemented with sodium pyruvate, nonessential amino acids, 10% fetal bovine serum, and 1% penicillin/streptomycin. GCGR-MYC-FLAG plasmid was purchased from Origene Technologies, whereas 5KR-GCGR-MYC-FLAG plasmid was generated by replacing lysines at 169, 333, 406, 423, and 451 using a QuikChange^TM^ site-directed mutagenesis kit (Stratagene) as per the vendor's protocol. N-terminal MYC-tagged GCGR plasmid was kindly provided by Dr. Ladds (12). GFP-tagged Rab4a and Rab5a plasmids were provided by Dr. Marino Zerial (70). EGFP-Rab4a S22N (catalog no. 49476) was purchased from Addgene. Rab5aS34N was generated by using the QuikChange site-directed mutagenesis kit. USP33 WT and USP33-cys-his (CH) mutant plasmids have been described before (18). All plasmid constructs were verified by DNA sequencing. For the generation of stably expressing WT-GCGR-MYC-FLAG or 5KR-GCGR-MYC-FLAG lysine mutant receptor cell lines, cells at a confluence of 40–50% were transfected with the respective plasmids using Lipofectamine 2000™ (Thermo Fisher Scientific), as per the manufacturer's protocol. Stably transfected cells were initially selected by culturing in growth medium supplemented with 1 mg/ml G418 and later maintained in 400 μg/ml G418-containing growth medium.

### Immunoprecipitation and immunoblotting

HEK-293 or HEPG2 cells stably expressing WT-GCGR-MYC-FLAG or mutant receptor 5KR-GCGR-MYC-FLAG were serum-starved for 1 h prior to stimulation with glucagon. Cells were solubilized in ice-cold lysis buffer containing 50 mm HEPES (pH 7.5), 2 mm EDTA (pH 8.0), 250 mm NaCl, 10% (v/v) glycerol, and 0.5% (v/v) IGEPAL CA-630 supplemented with phosphatase and protease inhibitors (1 mm sodium orthovanadate, 10 mm sodium fluoride, 1 mm phenylmethylsulfonyl fluoride, 5 μg/ml leupeptin, 5 μg/ml aprotinin, 1 μg/ml pepstatin A, and 100 μm benzaminidine; all from Sigma–Aldrich and also with 10 mm *N*-ethylmaleimide, which inhibits cellular deubiquitinase activity and preserves receptor ubiquitination. Cell lysate samples were centrifuged at 13,000 rpm for 10 min at 4 °C, and protein concentration was measured using Bradford reagent for the resulting supernatant. 800–900 μg of supernatant were used to set up immunoprecipitation using FLAG M2 affinity beads and kept for end-over-end rotation at 4 °C for overnight. Subsequently, the samples were washed three times with lysis buffer to reduce nonspecific binding, and bound protein was eluted in 2× SDS sample buffer. The proteins were resolved on a 4–20% gradient gel and then transferred onto nitrocellulose membrane (0.2 μm; Bio-Rad) for Western blotting. Membrane blocking and incubation with secondary antibody utilized 5% (w/v) dried skim milk powder dissolved in TTBS (0.2% (v/v) Tween 20, 10 mm Tris-HCl (pH 8.0), and 150 mm NaCl), and washing of immunoblots was done with TTBS. All primary antibodies were diluted in 5% (w/v) BSA prepared in TTBS. Immunoblotted proteins were detected through enhanced chemiluminescence using Super Signal West Pico Plus reagent. Chemiluminescence signals were observed and acquired with a charge-coupled device camera system (Bio-Rad Chemidoc-XRS). Protein band quantification was performed with Image Lab^TM^ Software (Bio-Rad).

### Determination of cell-surface GCGR by ELISA

HEK-293 cells were transiently transfected with MYC-GCGR-mCherry plasmid. 24 h after transfection, cells were serum-starved for 1 h prior to stimulation with 200 nm GCG for 15 and 60 min. After stimulation, cells were washed with cold PBS and incubated with anti-MYC (Cell Signaling Technologies) antibody (1:300 dilution in cold MEM, containing 0.1% BSA and 10 mm HEPES, pH 7.4), at 4 °C for 2 h with gentle side-to-side shaking. This was followed by washing with cold PBS and blocking with 2% BSA in PBS for 15 min. Cells were incubated with HRP-conjugated anti-rabbit secondary antibody at a dilution of 1:3000 for 1 h at 4 °C. Cells were then washed four times with cold PBS. TMB substrate solution warmed to room temperature was added for detection, and blue color development was stopped by adding 2 m H_2_SO_4_. The resulting absorbance was measured at 450 nm using BioTEK II Neo2S plate reader.

### RNAi

Nontargeting control siRNA and siRNA targeting USP33 (18), STAMBP (48), USP20 (18), USP14, and USP10 were purchased from Dharmacon GE Healthcare Life Sciences as described previously. The sequences of siRNA oligonucleotides are as follows: control nontargeting sequence, 5′-AAUUCUCCGAACGUGUCACGU-3′; USP33 (human), 5′-GAUCAUGUGGCGAAGCAU-3′; USP14 (human), 5′-GAGUAUUGCAACAGAAAUU-3′; USP10 (human), 5′-GGUGGCCUAUGUGGAAACUAAGUAU-3′; USP20 (human), 5′-CGUCGUACGUGCUCAAGAA-3′; STAMBP (human), 5′-UUACAAAUCUGCUGUCAUUUU-3′.

Early passage cells at a confluence of 40–50% were transfected with 20 μg of siRNA using Lipofectamine 2000^TM^ in a serum-free medium. Four hours after transfection, cells were transferred to complete culture medium and maintained for 48 h at 37 °C before assay procedures.

### Confocal microscopy

HEK-293 cells stably expressing WT-GCGR-MYC-FLAG or 5KR-GCGR-MYC-FLAG were transiently transfected with GFP-tagged Rab4a or GFP-tagged Rab5a. 24–48 h post-transfection cells were seeded on poly-d-lysine–coated 20-mm glass bottom plates. Cells were serum-starved for 1 h and stimulated with 1 μm GCG on the next day. After stimulation, cells were fixed with 5% formaldehyde diluted in Dulbecco's PBS (DPBS) for 20 min. Next, cells were permeabilized for 20 min with 0.1% Triton X-100 and subsequently incubated in primary antibody for MYC tag, 9E10 (Santa Cruz Biotechnology) at 4 °C overnight, followed by secondary antibody incubation at room temperature for 1–2 h. Cells were washed after cell fixation and antibody incubations with DPBS, whereas DPBS containing 2% BSA was used for making permeabilizing solution and antibody dilutions. Confocal images were captured with LSM-510 META confocal microscope with filter settings for the respective fluorophores; excitation was at 488 nm (GFP) and 561 nm (Alexa 594). Images acquired by using the ZEISS, ZEN imaging software were analyzed with ImageJ software and the JACoP plugin (71) to quantify fluorophore colocalization.

### Statistical analyses

Data are presented as means ± S.E. from technical replicates indicated in the figure legends. All of our experiments were conducted with a minimum of two separate lines of stably transfected cells. The type of statistical analysis and post-hoc test used are included in each figure legend. Our methods employed GraphPad PRISM version 8.3 (GraphPad Inc.), and a *p* value of <0.05 was considered significant.

## Data availability

All data, associated methods, and sources of materials are included in article or in the supporting information.
