# Supplementary material for: Agonist-activated glucagon receptors are deubiquitinated at early endosomes by two distinct deubiquitinases to facilitate Rab4a-dependent recycling
Source: J Biol Chem. 2021 Jan 13;295(49):16630–42. doi: 10.1074/jbc.RA120.014532 (PMC7864061; doi:10.1074/jbc.RA120.014532)
Supplement: Supplementary file 1 [file mmc1.pdf]

ONLINE SUPPORTING INFORMATION

Agonist-activated glucagon receptors are deubiquitinated at early endosomes by two distinct deubiquitinases to facilitate Rab4a-dependent recycling

Running Title: GCGR recycling is choreographed by USP33 and STAMBP

Suneet Kaur[1], Yuqing Chen[1], and Sudha K. Shenoy[1,2,3]

[1], Department of Medicine, Division of Cardiology, Duke University Medical Center, Durham, NC 27710, USA.
[2], Department of Cell Biology, Duke University Medical Center, Durham, NC 27710, USA
[3], Address correspondence to skshenoy@dm.duke.edu; Duke University Medical Center
Rm 148A, CARL Bldg. PO Box 102146; 213, Research drive, Durham, NC-27710
Phone: 919-681-5061

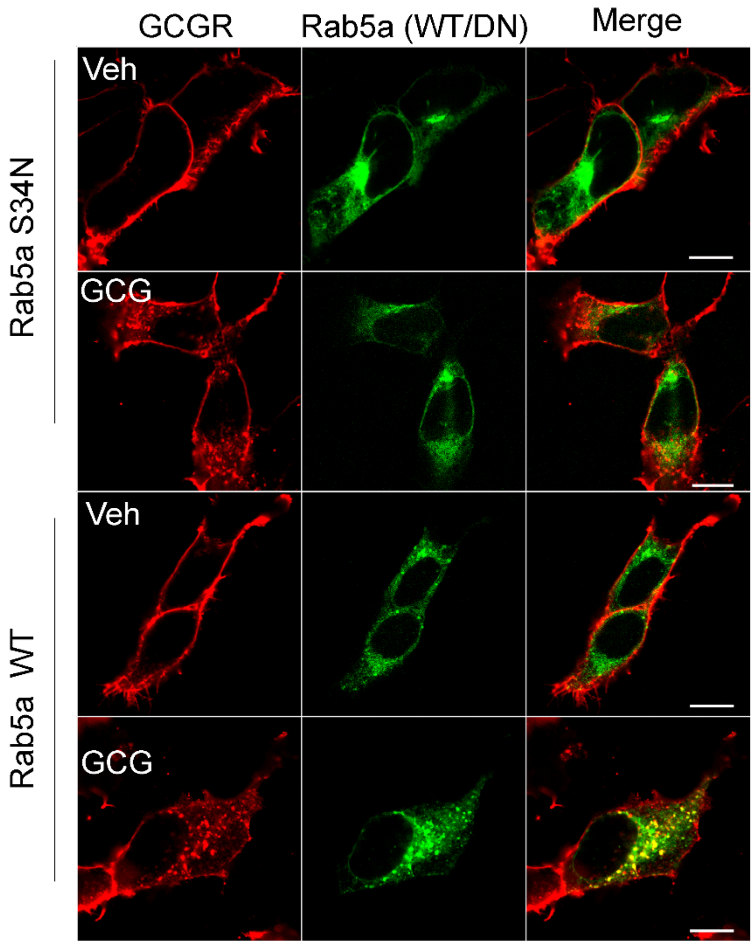

Supplementary Figure1: **Rab5a WT but not DN colocalizes with internalized GCGRs.** HEK-293 GCGR-MYC-FLAG stable cells transiently transfected with GFP-tagged Rab5a WT or Rab5a S34N (DN) were serum-starved for 1 h and then stimulated with 1 uM GCG for 15'. Cells were fixed, permeablized and immunostained for GCGR, using anti-MYC primary antibody (9E10, Santa Cruz). Confocal images for GCGR are shown in red (Alexa 594) and in green for Rab5a WT or Rab5a S34N as visualized using 510 LSM Meta confocal microscope. Confocal images shown are representative of two independent experiments. Scale bar = 10 µm.

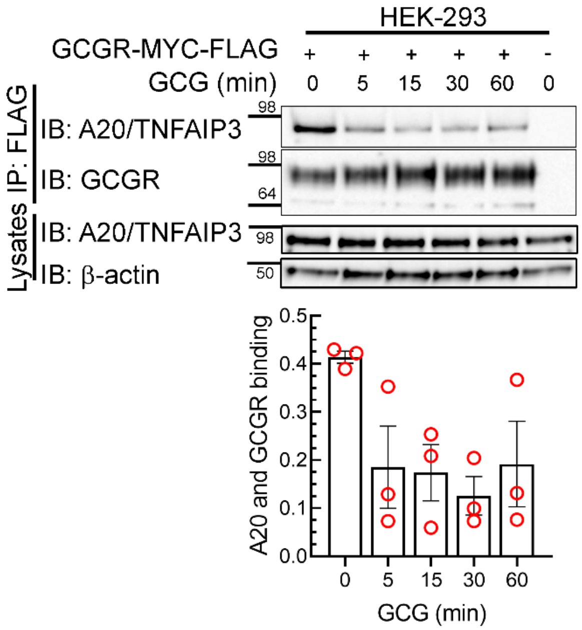

Supplementary Figure 2: The ubiquitin editing DUB A20 dissociates from activated GCGR. HEK-293 cells stably expressing GCGR-MYC-FLAG receptor serum starved for 1 h and stimulated with 200 nM GCG for the indicated times. Followed by immunoprecipitation with anti-FLAG M2 affinity agarose gel. The immunoprecipitates were resolved on 4-20% Tris-Glycine gels and immunoblotted for the indicated proteins. Bar graphs summarizes quantification of A20 normalized to receptor level from three independent experiments.

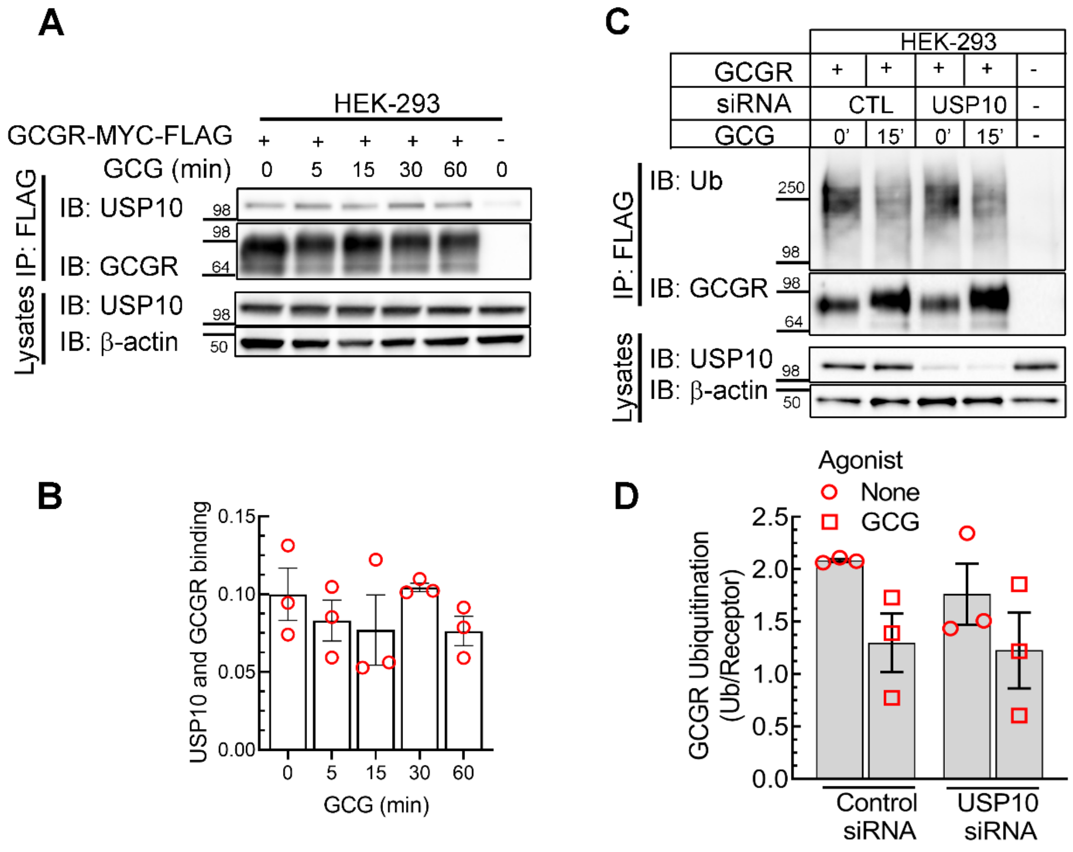

Supplementary Figure 3: The endosomal DUB USP10 does not serve as a cognate deubiquitinase for the GCGR. (A) HEK-293 cells stably expressing GCGR-MYC-FLAG were serum starved for 1 h and stimulated with 200 nM GCG for the indicated times followed by immunoprecipitation with anti-FLAG M2 affinity agarose gel. The immunoprecipitates were resolved on 4-20% Tris-Glycine gels and immunoblotted for the indicated proteins. **(B)** Quantification of USP10 normalized to receptor level is summarized from three independent experiments. **(C)** HEK-293 cells with stable expression of GCGR were transfected with either control siRNA or siRNA targeting USP10. Cells were serum starved for 1 h and stimulated with 200 nM GCG, followed by immunoprecipitation using anti-FLAG M2 affinity agarose gel and probed serially for Ub and GCGR. Bottom panels show lysates probed for USP10 and GAPDH. **(D)** Ubiquitin smear in each lane was normalized to the respective GCGR band and plotted as ratio from 3 independent experiments.

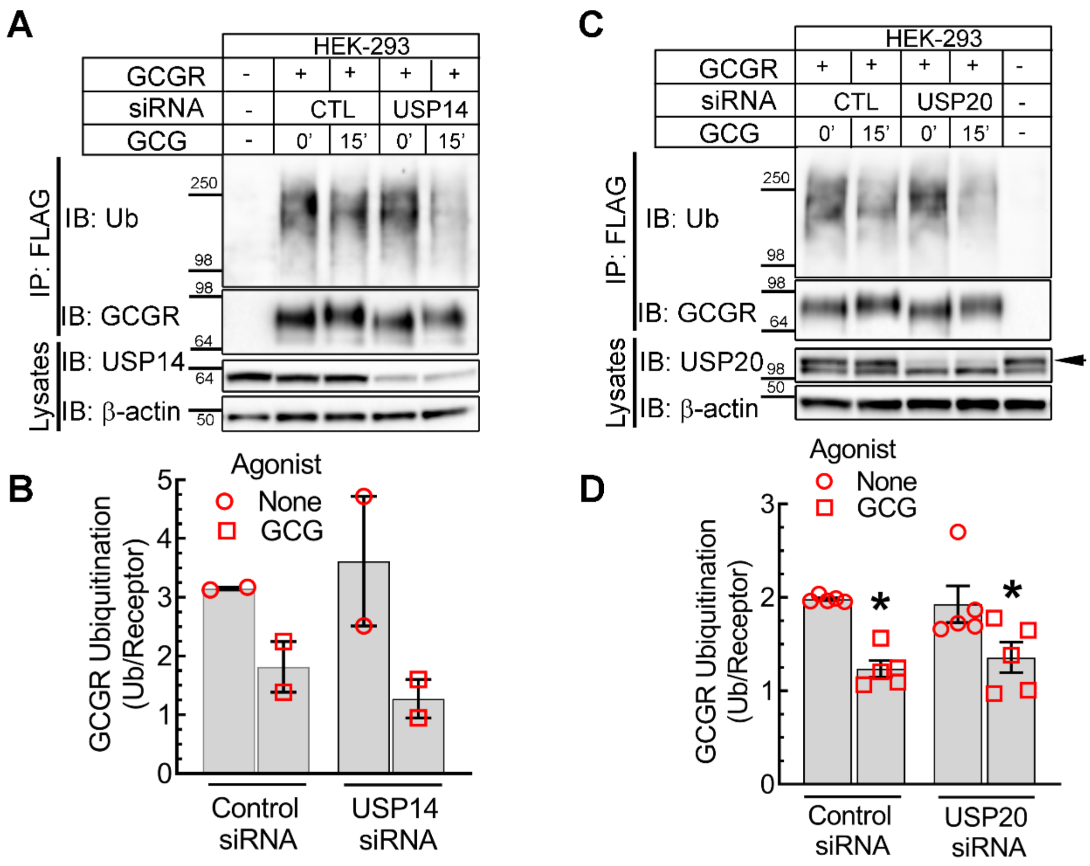

Supplementary Figure 4: USP14 and USP20 do not deubiquitinate the GCGR. **(A and C)** HEK-293 cells with stable expression of GCGR were transfected with either control siRNA or siRNA targeting USP14 (A) or USP20 (C). Cells were serum starved for 1 h and stimulated with 200 nM GCG, followed by immunoprecipitation using anti-FLAG M2 affinity agarose gel. **(B and D)** Ubiquitin smear in each lane was normalized to the respective GCGR band and plotted as ratio. Graphs show means ± S.E.M from two (USP14) and five (USP20) independent experiments. *, $p < 0.05$ versus NS conditions, two-way ANOVA, and Holm-Sidak’s multiple comparison test.

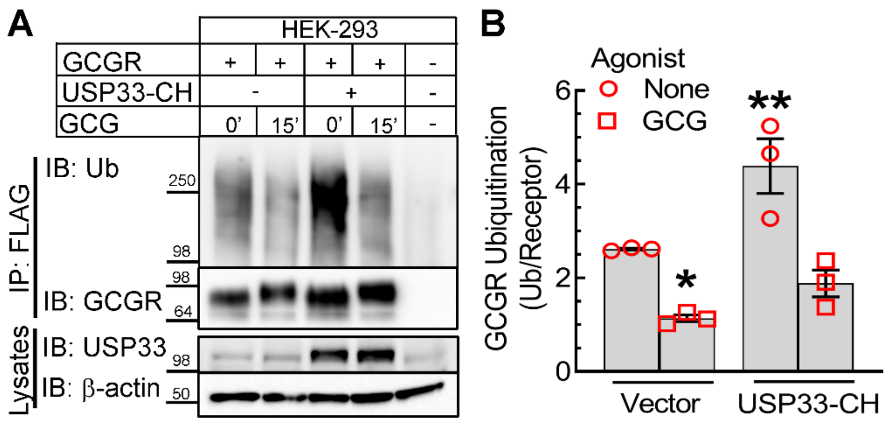

Supplementary Figure 5: Catalytically inactive USP33 increases basal ubiquitination of the GCGR in HEK-293 cells. (A) Experiment was conducted as in Figure 7A except that vector and USP33-CH mutant plasmids were transfected instead of siRNA. **(B)** Quantitation of GCGR ubiquitination was conducted as in Figure 1. The graph summarizes means ± S.E.M. from three independent experiments. *, $p < 0.05$ versus vector, no agonist, **, $p<0.05$ versus the rest, two-way ANOVA, and Holm-Sidak's multiple comparison test.
